# J. Houghton

**Published:** 1823-10

**Authors:** 


On the 14th of September, J. HOUGHTON, Esq. of Conduit-street.

